# Evaluating the Air Respiratory Capacity of *Awaous (Chonophorus) tajasica* (Gobiiformes: Oxudercidae): A Morpho‐Functional Study

**DOI:** 10.1002/jez.70084

**Published:** 2026-03-18

**Authors:** João Pedro Trevisan, Diego Venturelli, Wilfried Klein, André Luis da Cruz

**Affiliations:** ^1^ Museu de Zoologia da Universidade de São Paulo São Paulo Brazil; ^2^ Departamento de Biologia, Faculdade de Filosofia, Ciências e Letras de Ribeirão Preto Universidade de São Paulo Ribeirão Preto São Paulo Brazil; ^3^ Instituto de Biologia Universidade Federal da Bahia Salvador Bahia Brazil

**Keywords:** air bubbles, epithelial cells, gill ventilation, oxygen consumption, palate, vascularization

## Abstract

Surface air‐gulping behavior has been reported in several gobiid fishes, but its contribution to oxygen uptake remains unclear. This study aimed to evaluate the air‐breathing capacity of *Awaous tajasica* through behavioral observations, measurement of oxygen consumption, and analysis of the functional morphology of its palate and gills. Behavioral observations demonstrated that under hypoxic conditions, the fish moved to the water surface to engulf air bubbles, which remained within the buccal cavity during aquatic gill ventilation before being expelled through an opercular cavity. Oxygen consumption analysis revealed that *A. tajasica* obtained approximately 70% of its oxygen from the water and 30% from atmospheric air when under low aquatic oxygen conditions. Palate morphology did not reveal specialized respiratory structures, and only typical sensory structures such as taste buds were observed, indicating the absence of a dedicated aerial exchange surface. The retention of an air bubble in the buccal cavity during low aquatic oxygen concentration supports a functional contribution of air gulping to oxygen uptake in *A. tajasica*, demonstrating aerial oxygen acquisition without specialized air breathing organs in this gobiid species.

## Summary

- *Awaous tajasica* displays facultative air breathing under hypoxic conditions.
- Air bubbles are retained in the buccal cavity during aquatic gill ventilation.
- Approximately 30% of oxygen is obtained from air when water oxygen is limited.
- The palate lacks specialized respiratory morphology but has abundant taste buds.
- Combined morphological and behavioral evidence supports aerial respiration in this species.

## Graphical abstract

We demonstrated that *Awaous tajasica* performs facultative air breathing under experimental conditions of dissolved oxygen limitation, retaining air bubbles in the buccal cavity during gill ventilation. Morpho‐functional evidence indicates the use of atmospheric oxygen as a respiratory support under conditions of low water oxygenation.

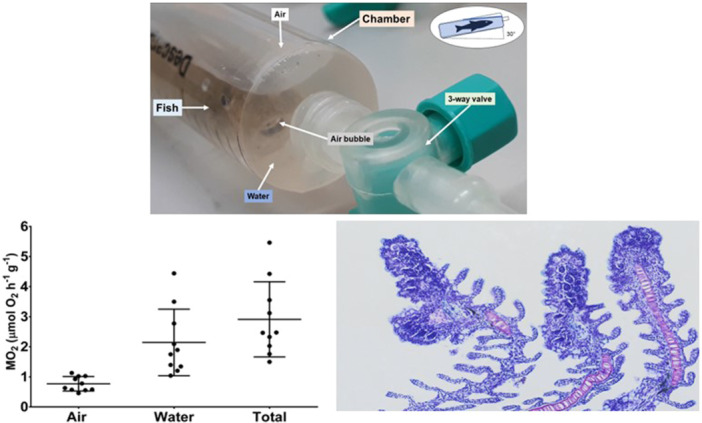

## 1 Introduction

Air‐breathing fishes inhabit various environments, including coastal rivers, marine and freshwater habitats, in both tropical and temperate regions. However, the most specialized air‐breathing fishes are typically found in tropical freshwater ecosystems, where seasonal droughts and low oxygen levels have led to diverse adaptations for aerial respiration (Graham 1997). Although many fish lack specialized air‐breathing organs, some can extract oxygen through permeable body surfaces when there is a gas diffusion gradient. This adaptability highlights the wide range of evolutionary strategies fish use to obtain oxygen in different aquatic settings (Nakamura 1994; Graham 1997).

The Gobiidae family, or gobies, is the second most diverse fish family with 258 genera distributed in various tropical environments (Eschmeyer and Fong 2023; Parenti and Thomas 1998). Traditionally, it was divided into five subfamilies: Amblyopinae, Gobiinae, Gobionellinae, Oxudercinae, and Sicydiinae (Pezold 1993). Recent revisions have reclassified it into two families: Gobiidae and Oxudercidae (Gill and Mooi 2012; McCraney et al. 2020). Oxudercidae now includes species from the former subfamilies and some European sand gobies previously in Gobiinae, with mudskippers being considered the ancestors of all Oxudercidae gobies. Such phylogenetic positioning would imply air breathing capacity to be a plesiomorphic state for the family.

One of the most intriguing goby genera is *Awaous*, also known as the river goby. Its phylogenetic placement among the subfamilies remains uncertain (Birdsong et al. 1988; Larson 2001; McCraney et al. 2020), but the lineage in which *Awaous* is generally included, is considered the closest one related to the amphibious air breathing gobies (McCraney et al. 2020).

Within the Gobionellinae subfamily, members exhibit a wide range of life habits (Larson 2001), being common for Gobionellinae gobies to gulp air bubbles, providing positive buoyancy to their heads during aquatic surface respiration in hypoxic events. Additionally, some of these gobies can extract oxygen from the engulfed air bubbles (Todd and Ebeling 1966; Gee and Gee 1995). *Gobionellus*, another member of the Gobionellinae subfamily, possesses a thin epidermal tissue layer in its palate, which could facilitate oxygen exchange and potentially serve as an air‐breathing surface (Aguilar et al. 2018). Air‐breathing is frequent in the Oxudercidae family (*sensu* McCraney et al. 2020) and members of the Oxudercinae and Ambliopinae subfamilies are well known for their air‐breathing abilities, observed in species like mudskippers (Taylor et al. 2005) and eel gobies (Gonzales et al. 2006; Gonzales et al. 2008). Members of the Sicydiinae subfamily, on the other hand, do not possess this air‐breathing capacity, being comprised of genera with highly specialized life habits, known for their efficient climbing abilities, typically found in fast‐moving waters and even waterfalls (Kido 1996).

Given *Awaous*’ uncertain phylogenetic placement, investigating its capacity for air breathing becomes particularly intriguing. In *Awaous transandeanus*, observations revealed aquatic surface respiration (ASR) as the fish moved toward the water's surface when exposed to hypoxic conditions (Kramer 1983). However, this author also noted that air breathing occurred only sporadically and was not consistently expressed among individuals. This finding underscores the importance of conducting further studies to gain a more comprehensive understanding of the air‐breathing capacity within this genus. Another intriguing aspect concerning *Awaous* is the presence of a complex arrangement of papillae observed in the oral and branchial cavities. Despite some previous investigation regarding these papillae (Sabino and Castro 1991; Kido and Ha 1993; Watson 1996), the function of these papillae remains unknown and could potentially play a role during air breathing.

The Brazilian species *Awaous tajasica*, unlike some other air‐breathing fishes, inhabits coastal rivers and is commonly found in rapids, which are known for an abundance of oxygen. Additionally, this species has never been reported outside of the water. It lives close to the substrate, feeding on algae and small invertebrates, and rarely removes its mouth from the river floor (Sabino and Castro 1991).

Gobies comprise around 200 genera and approximately 1500 species, including several air‐breathing species, some of which can breathe air both in aquatic environments and during terrestrial excursions (Graham 1997). Belonging to a family with many air‐breathing species, we ask whether *A. tajasica* retains any faculty related to aerial respiration (Perry et al. 2019). Since no publication reported any characteristic air‐breathing organ or air breathing related behavior for this species, we investigated this question by monitoring the fish's behavior, measuring aquatic and aerial oxygen consumption, and examined its morphology to assess potential respiratory surfaces. Thus, this comprehensive approach will allow for a better understanding of the species’ respiratory capacity, if present.

## 2 Methods

### 2.1 Experimental Animals

*Awaous tajasica* (*n* = 12) were collected in the Indaiá stream in Ubatuba (decimal coordinates: −23.40, −45.06). Animals were moved to an aquarium in the Ictiology Laboratory of Ribeirão Preto (Brazil), having been translocated in barrels containing local water and a constant supply of oxygen by aquarium aerators. Fish were allocated in aquariums with 96 litters capacity, in well‐aerated water. Temperature was kept at 25°C. Animals were fed every 2 days with commercial fish food rich in algae, mimicking the diet observed in the field (Sabino and Castro 1990). The acquisition and maintenance of fish were authorized by the Brazilian Biodiversity Authorization and Information System, Chico Mendes Institute for Biodiversity Conservation (SISBIO, ICMBIO protocol 74770‐1) and the Animal Use Ethics Committee at the University of São Paulo, Ribeirão Preto (protocol n° 20.1.498.59.0).

After the behavioral and oxygen uptake experiments, animals were euthanized in benzocaine solution (300 mg/L) and preserved in a buffer solution of formalin 10% with sodium bicarbonate and deposited in the collection of the Ribeirão Preto Ichthyology Laboratory (LIRP, USP).

### 2.2 Behavior

At the outset, we conducted an observational experiment to investigate the response of fish to conditions of low aquatic oxygen, particularly their tendency to approach the water's surface to either access the oxygen‐rich surface layer or to capture air bubbles. In this regard, we introduced a subgroup of five fish into an aquarium containing dechlorinated water. The animals were acclimated to the aquarium with normoxic water (PO_2_: 140 mmHg) for 40 min before starting the experiment. Then, a continuous injection of nitrogen was administered to gradually reduce the water's dissolved oxygen levels at a rate of 5 mmHg/min. To obtain data on water's PO_2_ and temperature, we employed two robust oxygen sensors (OXROB10) with optical detection principles (Redflash) along with an external temperature sensor (Pt100) connected to the FireStingO_2_ device (FSO_2_‐4, PyroScience). The fish were shielded from the observer and the entire procedure was documented using a camera (Sony Hybrid plus Handycam) to capture potential surfacing behavior and low oxygen exposure was interrupted when fish displayed behavioral signs of stress, such as erratic swimming, prolonged immobility, or exaggerated opercular ventilation. Fish were then returned to their maintenance aquarium for recovery and rested for at least a week before having their consumption of oxygen determined.

### 2.3 Measuring Aquatic and Aerial Oxygen Uptake

**Figure 1:**
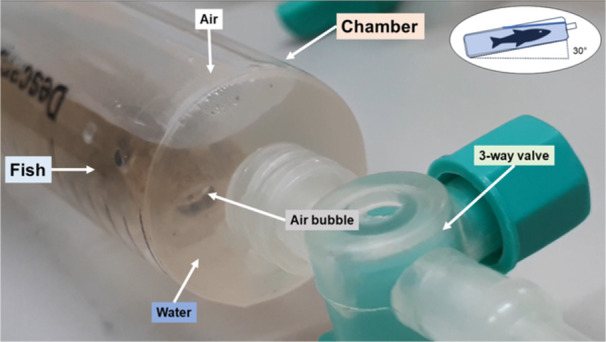
*Awaous tajasica* inside the respirometer at a 30‐degree inclination. Please note the presence of an engulfed air bubble inside the buccal cavity.

Oxygen consumption in air and water was measured in a closed respirometry system using a bimodal respirometer chamber at 26°C. Animals (*n* = 10; 2.48 ± 0.80 g) were not fed for 24 h and were individually transferred to respirometers. The respirometer consisted of a 20 mL capacity syringe, adjusted to a volume of 17 mL with 2 mL containing air and 15 mL containing water and fish. To account for the effective volume of water inside the respirometer, the fish's mass (assuming 1 g = 1 mL) was subtracted from the total water volume. The respirometer was positioned horizontally at an inclination of 30° (Figure 1), according to Gee and Gee (1995). This disposition was chosen based on the behavior presented during the aquarium‐simulated hypoxic event. The amount of water was enough to completely cover the animals but sufficient to permit access to air. To keep the system closed, a three‐way valve was placed at the top of the syringe, allowing the system to be opened and closed for sampling.

All fish were subjected to an acclimation period of 40 min in the respirometers before measurements. This period was established because preliminary experiments indicated that animals reached resting conditions within it, while a period exceeding 40 min could lead to significantly reduced water PO_2_ levels (40 mmHg). At the end of the acclimation period, the water in each respirometer was gently replaced with fresh air‐saturated water (normoxia) at the same temperature, taking care not to disturb the fish.

A sample of air and water was taken at the beginning (PiO_2_) and after 26.8 ± 0.6 min (PeO_2_) to evaluate the amount of oxygen consumed (ṀO_2_ = ΔPO_2_ x O_2_ solubility at 26°C; Dejours 1981) through water or air breathing. Respirometer volume was adjusted to compensate for the sample volume removed for measurement. Two flow cell sensors were used to measure PO_2_, one for water and another for air samples. These sensors were connected to an optical oxygen meter (FireSting, FSO_2_‐4, PyroScience), and calibration was performed before measurement according to instructions. Following measurements, the system was reopened to allow for the renewal of air and water. The oxygen consumption in both air and water was measured thrice. Two control treatments were used. One control employed the same normoxic water used in the animal respirometry trials. Due to ongoing oxygen consumption by microorganisms and possible chemical processes, the PO₂ decreased over time, potentially allowing O₂ diffusion from the air phase into the water. Furthermore, a second control was conducted using hypoxic water (PO_2_ = 13 mmHg) to quantify possible oxygen diffusion from air to water under low aquatic PO₂. Although minimal, these values were subtracted from the oxygen consumption measurements of the animals. These supplementary procedures were performed to validate aerial uptake measurements, since previous results were ambiguous (Kramer 1983), and since natural history, behavior, and previous respirometry analysis pointed to very weak aerial oxygen consumption. Subsequently, post‐experiment, all animals were weighed (±0.001 g) to standardize the O_2_ consumption rates by body mass.

We also conducted a linear regression analysis to evaluate the relationship between body mass and oxygen consumption, considering that metabolic rate is influenced by body size. The analyses and graphical representations were generated using GraphPad Prism software version 7. Data are presented as means ± SD.

### 2.4 Morphology

The palate, located on the roof of the buccal cavity, and the gill arches from the left side were embedded in paraffin and sectioned at 5 μm thickness. The analysis was performed using three fish, and images of the gills and palates were obtained from each individual. For the gills, 16 images per fish were analyzed, including eight stained with toluidine blue and eight with hematoxylin and eosin (H&E). For the palates, eight images per fish were analyzed, four stained with Masson's trichrome and four with H&E. The slides were examined in their entirety to ensure the selection of representative areas for image acquisition. The analyses focused on evaluating the pattern of cellular organization, considering characteristic histological features observed in the different structures and staining protocols. Histochemical analyses were also conducted using the periodic acid‐Schiff (PAS) reaction for neutral mucopolysaccharides, and Alcian blue staining at pH 1.0 for acidic mucopolysaccharides. All sections were examined under a light microscope (Olympus BX61VS) equipped with a digital camera (Olympus XM10).

The fixed arches from the right side were dehydrated sequentially in ethanol ranging from 30% to absolute concentration. They were then dried with a Leica CPD 030 critical point drier, mounted onto aluminum stubs using silver paint, and finally sputter‐coated with gold. Samples were analyzed using a JEOL JSM 6610LV scanning electron microscope.

## 3 Results

### 3.1 Behavior

For most of the experiment's duration, the animals remained at the bottom of the aquarium, except when the water reached a state of severe oxygen depletion (PO_2_ = 20 mmHg). Under such conditions, the animals displayed an inclination of the body to rise to the water's surface, with two individuals adopting an arched or vertical position against the aquarium wall. When the oxygen concentration dropped below 10 mmHg, all the animals exhibited surfacing behavior. In some instances, we observed air bubbles within the mouths of the fish following these surfacing events.

### 3.2 Aquatic and Aerial Oxygen Uptake

**Figure 2:**
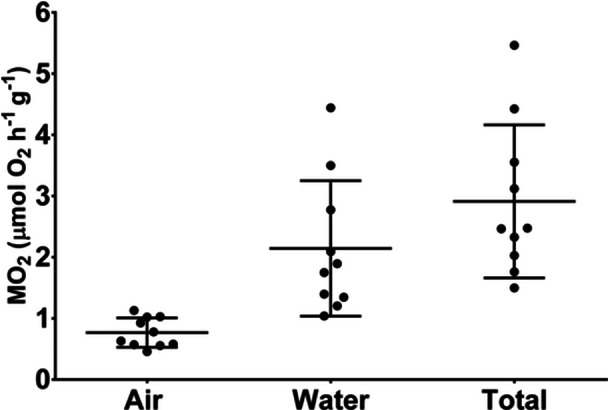
Aerial, aquatic and total mass‐specific O_2_ consumption rates in *Awoaus tajasica* measured at 26°C in a bimodal respirometer under normoxic exposition in both air and water phase. Each point represents the individual values (*n* = 10). The bar represents mean and standard deviation.

**Figure 3:**
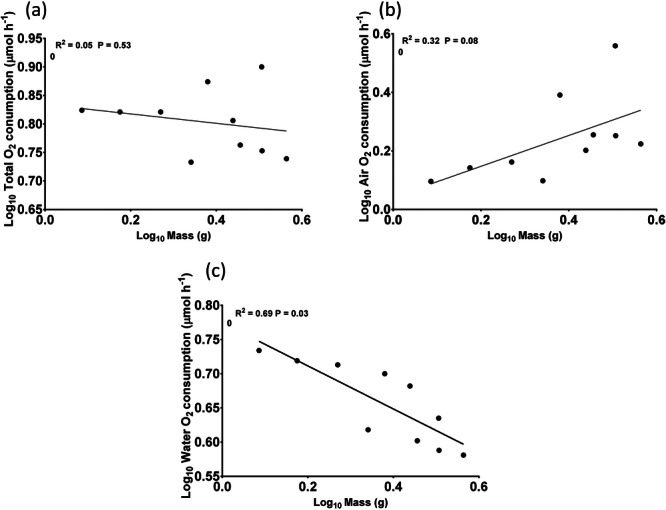
Linear regression between body mass and total (a), aerial (b) and aquatic (c) O_2_ consumption. The data were log‐transformed, and each point represents an individual value.

Total oxygen uptake was on average 2.91 ± 1.19 μmol O_2_ g^−1^ h^−1^. Although the main oxygen consumption occurred in the aquatic environment (2.14 ± 1.05 μmol O_2_ g^−1^ h^−1^), there was uptake of atmospheric oxygen (0.77 ± 0.23 μmol O_2_ g^−1^ h^−1^) (Figure 2). Variations in animal size contributed to an increased range in mass‐specific oxygen consumption (Table 1). While no correlation was observed between mass and total oxygen consumption (Figure 3a), an inversely proportional relationship emerged between mass and aquatic oxygen consumption, with smaller animals consuming more oxygen from water than larger ones (Figure 3b). Conversely, for aerial oxygen consumption, this correlation reversed, with larger animals consuming proportionally more aerial oxygen than smaller ones (Figure 3c). Even after subtracting the oxygen consumption of the animals from the low oxygen control values, the levels of oxygen consumed were still higher. Therefore, the drop in aerial oxygen was not caused by the diffusion of oxygen into the water.

**Table 1:** Mean values and standard error of all variables measured in 10 animals of *Awoaus* (*Chonophorus*) *tajasica.*

Variables	Mean ± SD
Body mass (g)	2.45 ± 0.76
PiO_2_ air (mmHg)	140.81 ± 1.74
PeO_2_ air (mmHg)	133.06 ± 2.99
PiO_2_ water (mmHg)	123.63 ± 30.08
PeO_2_ water (mmHg)	34.91 ± 10.01
MO_2_ total (μmol g^−1^ h^−1^)	2.91 ± 1.19
MO_2_ air (μmol g^−1^ h^−1^)	0.7 ± 0.23
MO_2_ air (% MO_2_ total)	28.13 ± 7.54
MO_2_ water (μmol g^−1^ h^−1^)	2.14 ± 1.05
MO_2_ water (% MO_2_ total)	71.87 ± 7.54

Abbreviations: e, final of experiment; i, initial of experiment; MO_2_, mass‐specific oxygen consumption rate, PO_2_, partial pressure of oxygen.

### 3.3 Morphology

#### 3.3.1 Palate Skin

**Figure 4:**
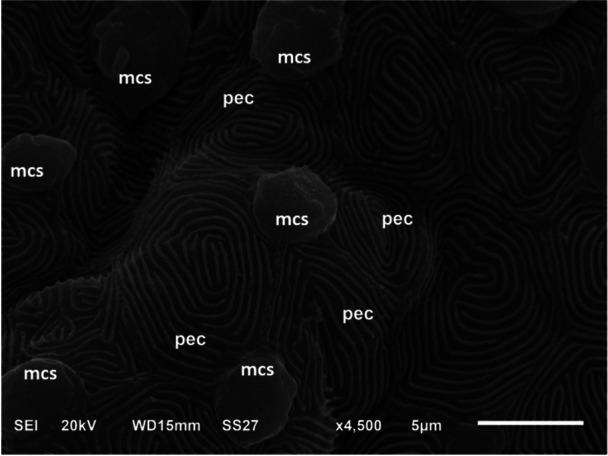
Scanning electron micrograph of the palate surface of *Awaous tjasica*, showing a mosaic arrangement of irregular polygonal epithelial cells (pec), bordered by evenly spaced microridges. Mucous cell secretions (mcs) were observed adhering to the microridges between the epithelial cells.

**Figure 5:**
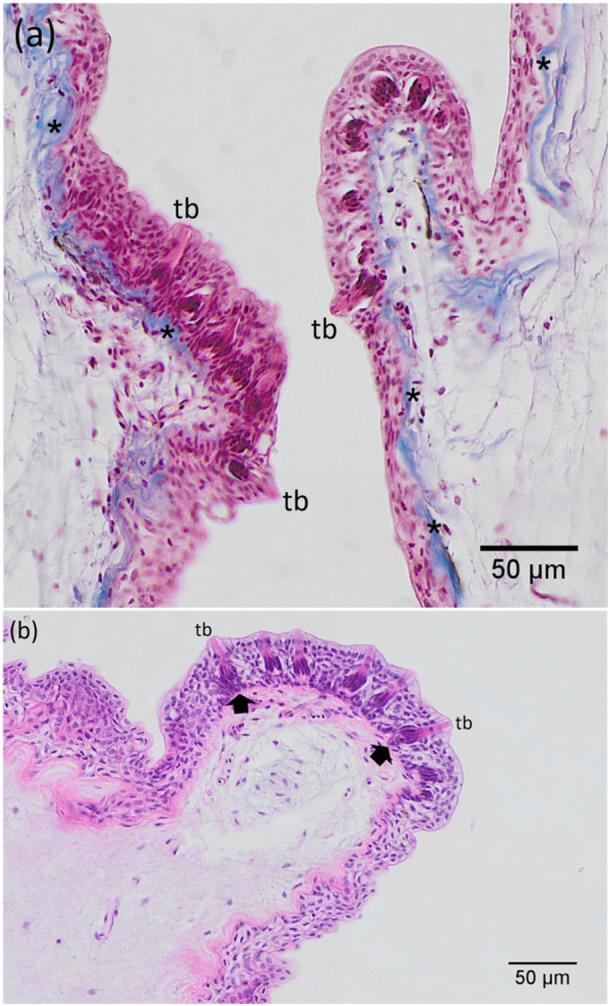
Light microscopy images of upper palate mucosa of *A. tajasica*. (a) The stratified squamous epithelium, stained with Masson's trichrome, covers the palate and presents oval mucous cells embedded in the superficial layer, supported by a layer of cuboidal epithelial cells and an inner layer of columnar cells. Immediately beneath the epithelium, the lamina propria (stained blue) is observed, composed of collagen fibers (*) that provide structural support. (b) Taste buds (tb), stained with hematoxylin and eosin, are distributed throughout the palate, exhibiting a distinct elongated cup‐shaped structure. The apical portion of the taste buds projects outward, while the basal part connects to nerve fibers (black arrows). The stratified squamous epithelium overlies the lamina propria, which contains collagen fibers (light pink) supporting the epithelium.

**Figure 6:**
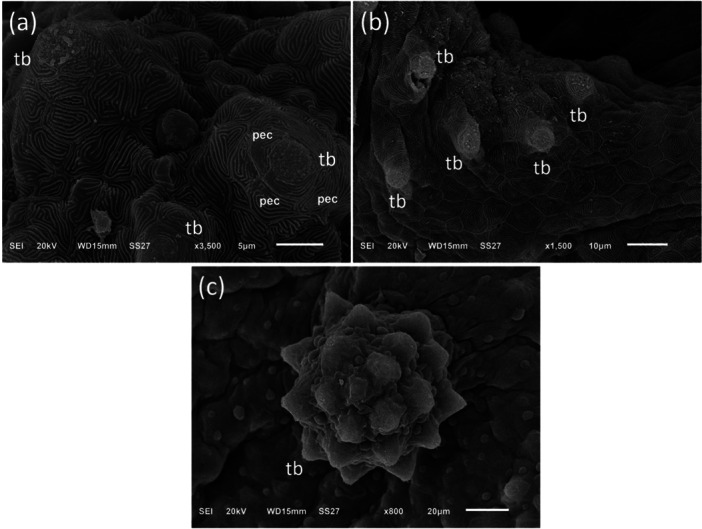
Scanning electron microscopy showing three distinct types of taste buds in *Awaous tjasica*: (a) a taste bud located between polygonal epithelial cells (pec), (b) a globular type also surrounded by polygonal epithelial cells, and (c) a rosette‐shaped type, both extending above the epithelium surface.

The palate is a part of the upper jaw, being approximately U‐shape. Its surface exhibited a mosaic pattern of irregular polygonal epithelial cells, delineated by evenly spaced microridges. Mucous cell secretions adhered to these microridges in between the epithelial cells (Figure 4). The palate was covered by a stratified squamous epithelium, featuring oval mucous cells embedded in its superficial layer. The mucosa of the upper palate consisted of a stratified epithelium supported by a layer of cuboidal epithelial cells and an inner layer of columnar epithelial cells (Figure 5a). Occasionally, few blood capillaries have been observed in the epithelium. Among the epithelial cells, predominantly, taste buds were distributed throughout the palate. They exhibited a distinct elongated cup‐shaped structure located at the edge of the epithelial cell layer. These taste buds had their apical part projecting outward and their basal part connecting to nerve fibers (Figure 5b). There were three types of taste buds present: one limited to the level of the epithelium, a globular and a rosette type, which extended above the epithelium (Figure 6a–c).

#### 3.3.2 Gills

**Figure 7:**
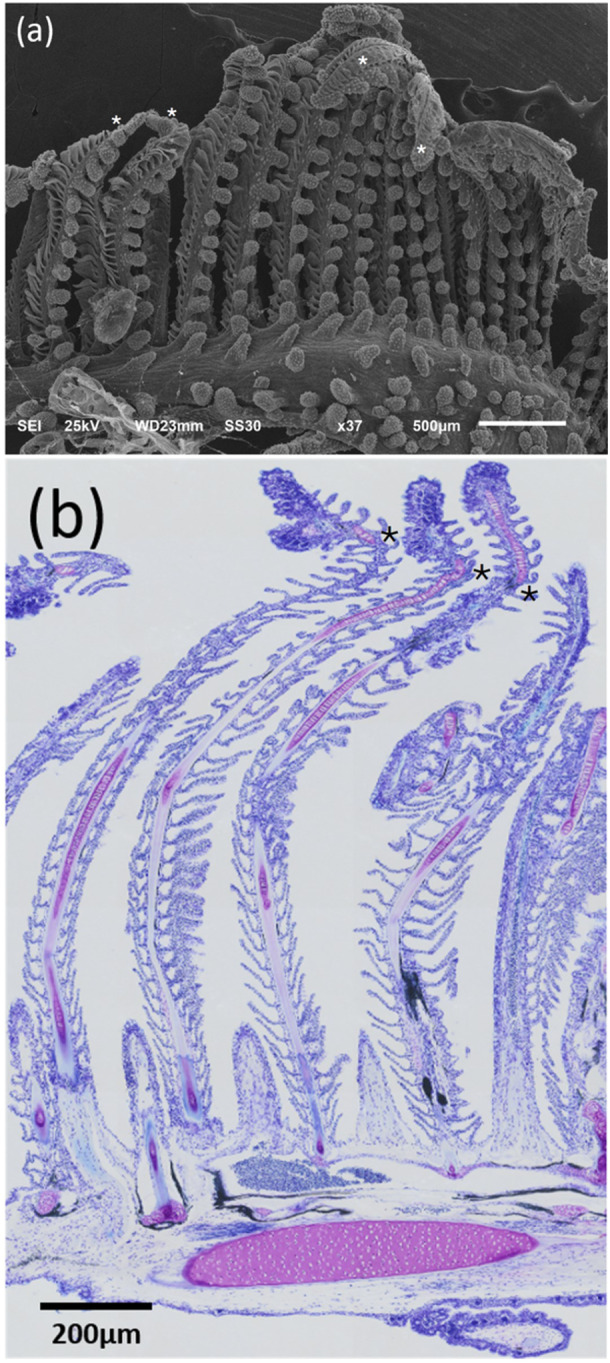
Gill arches of *Awaous tajasica*, composed of rows of filaments with bending and/or twisting (*) at the apex, as observed in (a) scanning electron microscopy and (b) light microscopy stained with toluidine blue.

The gills of *A. tajasica* consisted of four gill arches on each side of the head, each arch composed of two rows of filaments forming the hemibranchs, and one pseudobranch on each side of the head. These filaments may exhibit bending and slight twisting (Figure 7a,b). In the filamentary epithelium, the pavement epithelial cells presented microridges on their surfaces, which were in direct contact with water. Among these cells, ionocytes, also known as mitochondria‐rich cells or chloride cells, were present, as well as mucous cells. Ionocytes were primarily located in the filamentary epithelium near the base of the lamella and at the base of the lamellar epithelium. In the secondary lamella, some ionocytes were noticeable at the transition with the gill filament.

**Figure 8:**
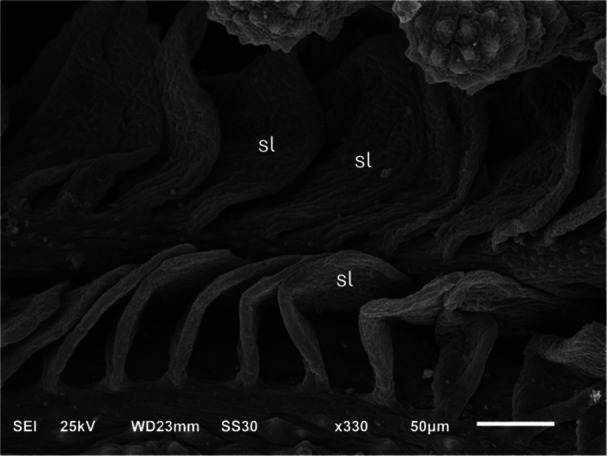
Scanning electron micrograph of a gill filament of *Awaous tajasica* showing the emergence of secondary lamellae (sl), which exhibit a rectangular shape, smooth surfaces, and a wavy appearance.

**Figure 9:**
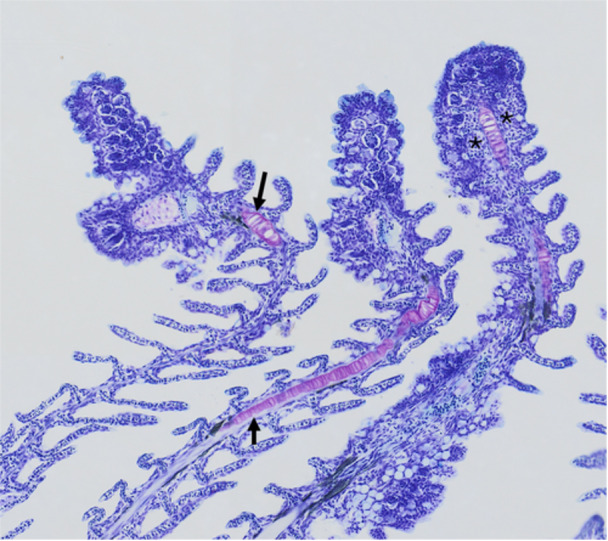
Light microscopy stained with toluidine blue showing gill filaments with cartilaginous tissue (arrows), stained violet, extending from base to apex. Numerous blood spaces containing abundant blood cells (asterisks) are observed in the apical region.

**Figure 10:**
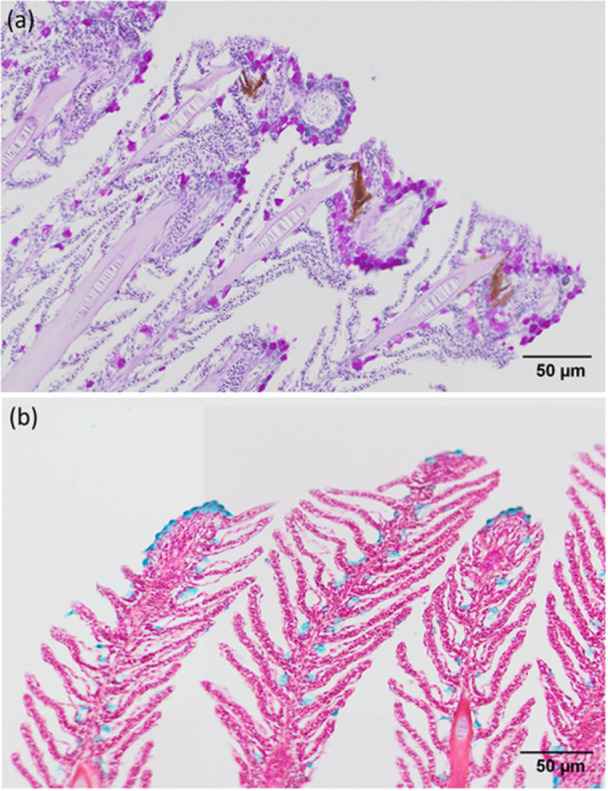
Light micrographs of the gill lamellar epithelium of *Awaous tajasica*, stained with (a) Periodic Acid‐Schiff (PAS), showing mucous cells positive for acidic mucopolysaccharides in magenta, and (b) Alcian Blue, showing mucous cells positive for neutral mucopolysaccharides in blue.

The secondary lamellae were composed of squamous epithelial cells that cover the surface, and their boundaries were distinctly defined by continuous microridges. Each gill filament gave rise to secondary lamellae, which showed a rectangular shape, smooth surfaces, and a wavy appearance (Figure 8). Internally, cartilaginous tissue was present from the base to the apex of the gill filaments. Additionally, numerous blood capillaries with abundant blood cells were present in the apical region of the gill filaments (Figures 7b and 9). Similarly, to the filamentary epithelium, mucous cells were observed in the lamellar epithelium which revealed positive PAS and Alcian blue, indicating the production of acidic and neutral mucopolysaccharides (Figure 10a,b).

## 4 Discussion

Within the genus *Awaous*, *A. transandeanus* was observed performing aquatic surface respiration under hypoxic conditions, with aerial respiration showing a weak or inconsistent response (Kramer 1983). Our study investigated atmospheric oxygen uptake through behavioral observations in aquariums and oxygen consumption under decreased oxygen conditions, alongside the morphology of palate epithelium and gills in *A. tajasica*. We demonstrate that, even inhabiting environments with good oxygen availability, *A. tajasica* has the ability for aerial respiration.

During conditions of low aquatic oxygen, animals moved to the surface to reach for an air bubble to be engulfed into the buccal cavity. Since we performed this procedure with a group of five animals, a social component of surfacing together to reduce the risk of predation might have influenced the fish's behavior, but proper behavioral studies are needed to verify such a response for *A. tajasica*. The ingestion of an air bubble, however, is a common behavior, being observed in several gobies closely related to *Awaous* (Kramer 1983), and aquatic surface respiration is a common response of fish during hypoxic events, and not necessarily related to air breathing, despite accidental ingestion of air bubbles. While we did not consistently quantify gill ventilation, progressive hypoxia seemed to increase opercular frequency in all fish exposed to hypoxia, but only at aquatic PO_2_ below 20 mmHg surfacing behavior and bubble capture were executed to supplement the animals’ oxygen demands. Moreover, we also noticed a pronounced vascularization in the gill region, characterized by a reddish appearance. Such observations preceded the onset of ASR and suggest an increase in both ventilation and perfusion of gills to increase oxygen absorption. During ASR, animals adopted an arched or vertical positioning, trapping air bubbles in the buccal cavity. The air bubble remained within the buccal cavity for some time, being in contact with the palate before being expelled through the opercular cavity of one side, without remaining in contact with the gills for a significant amount of time (see Material S1). Gee and Gee (1991) studied five species of Eleotridae and fifteen species of Gobiidae subjected to progressive hypoxia and found that all fish used ASR and maintained a bubble in their buccal cavity. In all species extensively utilizing the buccal bubble, gill ventilation rate decreased significantly as the percentage of fish using ASR increased. They noted that when water was inspired, the bubble moved backward. Upon expiration of buccal water, the bubble moved forward but was contained in the buccal chamber by buccal flaps that closed off the mouth. The absorption of dissolved oxygen provided by the bubble was observed in *Carassius auratus* subjected to hypoxia, resulting in an increase in arterial O_2_ pressure, i.e., a combination of oxygenated surface water with inspired atmospheric air ensured oxygen transport in the blood for the maintenance of aerobic metabolism in severely hypoxic water (Burggren 1982). Thus, the bubble could be maintained with the fish in various positions, and water, upon inspiration, flowed over a large portion of the bubble surface. The air bubble maintained in the mouth of *A. tajasica* might act in a similar manner as seen in *C. auratus*.

The same authors also discussed that the bubble appeared to provide slight positive buoyancy for the head in the arched position and for the body in the vertical position, as part of the snout often emerging, positioning the mouth at the water surface, which also seems to favor *A. tajasica'*s lack of a swim bladder. The buccal bubble furthermore has been suggested (Gee and Gee 1991) to increase dissolved oxygen in the buccal chamber before ventilating the gills by oxygen diffusing from a high concentration in the bubble to the hypoxic water. Some species that gulp air bubbles may live in open areas with organic debris, among rocks, or in vegetation. They use these environments and their arched position during aquatic surface respiration (ASR) to avoid potential predators (Chapman and McKenzie 2009).

Regarding oxygen consumption, *A. tajasica* obtained approximately 70% of its oxygen from water and 30% from atmospheric air in the respirometer setup. Similarly, in *Gobiodon histrio*, a consumption of about 60% of oxygen from water and 40% from atmospheric air was observed (Nilson et al. 2004). For *G. histrio*, tolerance to air exposure seems to reflect the importance of its coral associated lifestyle, regardless of nocturnal hypoxia and periodic exposure to air during low tides. On the other hand, *A. tajasica*, despite inhabiting rivers with apparently adequate oxygen levels, may also show hypoxia tolerance due to its bottom‐dwelling habits, since *A. tajasica* feed during the day by excavating the substrate and engulfing sediments, while remaining semi‐buried in the substrate during the night (Sabino and Castro 1990).

The largest animals presented higher aerial oxygen consumption, showing that aerial respiration is positively related to animal size. This result could be explained with larger animals demanding more oxygen in absolute quantities, but the gill surface area among fish normally shows a scaling exponent of less than 1 (Hughes 1984). This leads to a situation in larger fish possessing a lower exchange surface area, and larger *Awaous* might compensate for oxygen demand by increasing aerial respiration. No morphological surface area data for *A. tajasica*, however, is available and the intraspecific scaling relations are unknown to this species.

Initially, we considered whether any structures of the palate could serve as respiratory surfaces. Our morpho‐functional study revealed that the palate of *A. tajasica* is covered by a stratified squamous epithelium with scarce blood vessels, in contrast to the highly vascularized palates of other gobies such as *Gobionellus oceanicus*, which show a high O₂ diffusion capacity and indicate respiratory potential (Aguilar et al. 2018). These structural characteristics indicate that the palatal epithelium is not specialized for gas exchange. Consistent with Sabino and Castro (1991), the epithelial protrusions of the palate are taste buds, similar to those present on the gill arches and filaments. Each taste bud extends above the epithelium, with apical microvillar extensions (taste hairs) opening through taste pores, and contains sensory cells throughout, connected to subepithelial sensory nerves (Fishelson and Delarea 2004; Mistri et al. 2016). According to Fishelson and Delarea (2004), types I and II taste buds protrude above the surrounding epithelium in lobules of various shapes, whereas type III terminates at the level of the epithelium. Three types of taste buds were identified in *A. tajasica*: one limited to the epithelium, a globular type, and a rosette type, which protrudes above the epithelium. Differences in density and distribution of taste buds likely reflect feeding strategies and diet, representing ecomorphological adaptations and not a respiratory function.

Since there were no typical air breathing organ and a respiratory surface in the buccal cavity with rich vascularization, we analyzed if the gills could occupy such a respiratory role. The gills of *A. tajasica* appear to be functionally suitable for aquatic life, possessing characteristic gill arches and secondary lamellae. We also observed that the gill filaments were notably reinforced with cartilaginous tissue from the base to the apex, providing them both with flexibility and mechanical resistance. This can reduce injuries caused by sediments entering through the mouth and subsequently being expelled through the opercular openings during gill ventilation. The support by cartilaginous tissue can also be important to prevent filament collapse, ensuring the maintenance of blood flow even if the gills do not play a significant role in aerial gas exchange (Gonzales et al. 2008). Turko et al. (2020), when evaluating calcification in the gill filaments, found that the morphology of gills is closely related to aerobic capacity and tolerance to environmental stressors, such as hypoxia.

The morphological design of the gills of *A. tajasica* also deserves attention, especially due to the large size of the marginal channels of the secondary lamellae. Similar structures were described by Al‐Kadhomiy and Hughes (1988) in the mudskipper *Boleophthalmus boddarti*, where the enlargement of the outer channel facilitates gas exchange with air when the fish is out of water. Although we did not specifically analyze the vascularization of the gills in *A. tajasica*, the notably wide marginal channels, which appear capable of holding a larger volume of blood, may serve as an important site for oxygen absorption under hypoxic conditions. This feature is likely relevant for gas exchange both in water—enhancing oxygen uptake from the aquatic environment—and potentially when air is used incidentally or facultatively during severe hypoxia episodes.

In addition to these vascular and structural features, the presence of gill filaments exhibiting gentle curvatures and slight twisting may represent structural adaptations to aerial respiration, commonly observed in amphibious or facultative air‐breathing fish species (Low et al. 1988). Such morphological features can help prevent gill collapse outside water, maintain moist microenvironments between filaments, support gas exchange under aerial conditions, and reflect phenotypic plasticity in response to environmental stressors. Some studies have documented ramified filaments, fused lamellae, and reinforcement structures that resist coalescence during emersion (Tamura et al. 1976; Daxboeck and Heming 1982; Wilson et al. 1999). These structural arrangements create a three‐dimensional geometry that stabilizes the gills in the absence of buoyant support, enabling partial of branchial function during air breathing. The observation of curved and slightly twisted filaments in our specimens may therefore indicate a functionally equivalent mechanism, supporting the hypothesis that subtle morphological modifications contribute to facultative air‐breathing capacity in *A. tajasica*.

Another aspect observed in the gills of *A. tajasica* was the histochemical staining with PAS and Alcian Blue. This staining evidenced the production of neutral, acidic and sulfated mucosubstances (glycoproteins) by the mucous cells, notably in the lamellar epithelium that lines the marginal channels. Similar observations were made in the air‐breathing fish *Hoplerythrinus unitaeniatus* (Moron et al. 2009). In fish, neutral mucosubstances may protect and lubricate the gill epithelium against injuries (Sibbing and Uribe 1985), while acidic and sulfated mucosubstances prevent pathogen proliferation (Mittal et al. 1994). In the case of air‐breathing fish, acidic and sulfated mucosubstances are related to protection against desiccation during exposure to atmospheric air (Parashar and Banerjee 1999; Chandra and Banerjee 2003). In this way, such aspect would be another suggestion of the capacity for aerial respiration in *A. tajasica*.

## 5 Conclusions

The present study advances the understanding of the respiratory biology of *A. tajasica* and highlights the importance of evaluating events along the oxygen cascade to interpret behavioral and physiological responses to environmental hypoxia. The ability to ingest and retain air bubbles within the buccal cavity may provide an adaptive advantage to this facultative air‐breathing species, particularly in larger individuals exposed to fluctuating oxygen availability. Morphological analyses of candidate respiratory surfaces revealed no specialized extra‐branchial respiratory organ. Instead, the observed structural features indicate that atmospheric oxygen uptake occurs across the branchial lamellae. These findings demonstrate that *A. tajasica* employs a respiratory strategy combining aquatic respiration with facultative aerial gas exchange mediated by the gills. Considering the phylogenetic position of *Awaous*, the occurrence of surface breathing behavior under hypoxic conditions, together with gill and palate morphology, suggests the retention of a conserved respiratory capacity rather than the evolution of a novel air‐breathing organ, consistent with patterns described in other goby species.

## Conflicts of Interest

The authors declare no conflicts of interest.

## Supporting information

bubble capture slow motion.mp4.

air bubble in mouth.mp4.

air bubble in mouth slow motion.mp4.

bubble capture.mp4.

Supplement material.pdf.

## Data Availability

The data that support the findings of this study are available from the corresponding author upon reasonable request.
