# Supplementary material for: Evaluating the Air Respiratory Capacity of Awaous (Chonophorus) tajasica (Gobiiformes: Oxudercidae): A Morpho‐Functional Study
Source: J Exp Zool A Ecol Integr Physiol. 2026 Mar 18;345(5):498–507. doi: 10.1002/jez.70084 (PMC13161352; doi:10.1002/jez.70084)
Supplement: Supplementary file 5 — Supplement material.pdf. [file JEZ-345-498-s002.pdf]

Evaluating the air respiratory capacity of *Awaous (Chonophorus) tajasica* (Gobiiformes: Oxudercidae): a morpho-functional study

João Pedro Trevisan, Diego Venturelli, Wilfried Klein, André Luis da Cruz

Journal of Experimental Zoology A

The four supplementary videos show:

- A fish capturing a bubble at the water surface (bubble capture'; 'bubble capture_slow motion') and the air bubble moving inside the buccal cavity;
- The air bubble remained within the buccal cavity for some time ('air bubble in mouth'; 'air bubble in mouth_slow motion'), being in contact with the palate before being expelled through the opercular cavity on the left side.
